# Effect of different smoking methods on the quality of pork sausages

**DOI:** 10.14202/vetworld.2018.1712-1719

**Published:** 2018-12-20

**Authors:** Debajit Bhuyan, Ankur Das, Saurabh Kumar Laskar, Durlav Prasad Bora, Shantanu Tamuli, Mineswar Hazarika

**Affiliations:** 1 Department of Livestock Products Technology, College of Veterinary Science, Assam Agricultural University, Khanapara, Guwahati - 781 022, Assam, India; 2 Department of Veterinary Microbiology, College of Veterinary Science, Assam Agricultural University, Khanapara, Guwahati - 781 022, Assam, India; 3 Department of Veterinary Biochemistry, College of Veterinary Science, Assam Agricultural University, Khanapara, Guwahati - 781 022, Assam, India

**Keywords:** liquid smoke, pork sausage, quality attributes, smoking

## Abstract

**Aim:**

An experiment was conducted to evaluate the nutritional, physicochemical, microbiological, and sensory attributes of pork sausages treated with conventional smoking (CS) and liquid smoke (LS).

**Materials and Methods:**

Pork sausages were prepared by employing CS (T_1_) and by addition of LS at 3% (T_2A_), 5% (T_2B_), and 7% (T_2C_) while smoking was not done in control (C) sausages. The ready-to-eat pork sausages were evaluated in terms of proximate composition, emulsion stability (ES), cooking loss (CL), pH, water activity (a_w_), texture profile analysis (TPA), and shear force on the day of preparation and the shelf life of the sausages was evaluated on the basis of thiobarbituric acid reactive substance (TBARS) value, organoleptic qualities, total viable plate count, total psychrophilic count, and yeast and mold counts at 5-day interval up to 15 days under refrigerated storage (6±1°C).

**Results:**

The mean percentage moisture and percentage ether extract contents of the conventionally smoked sausages (T_1_) exhibited significant difference (p≤0.01) with the rest of the formulations. However, in terms of mean percentage crude protein and percentage total solids, no significant difference (p≥0.05) was recorded between the treatment groups. The mean ES (ml of oil/100 g emulsion) of the different sausage emulsions ranged from 1.88 to 3.20, while the mean a_w_ values among the sausage formulations were found to be non-significant. In terms of mean percentage, CL and pH values, significantly lowest (p≤0.01) values were recorded by the T_1_ sausages. The mean TBARS values recorded at different periods of time in respect of all the treatment groups ranged from 0.10 to 0.33 mg malanoldehyde [MDA]/kg of sausages which are well within the permissible limit. The highest shear force values (KgF) were recorded by the sausages of T_1_ formulation (p≤0.01), while TPA of the sausages did not record any significant difference (p≥0.05) among the treatments. Organoleptic studies revealed acceptability of the sausages up to 10 days of refrigerated storage irrespective of treatments employed; however, the sausages of T_1_ formulation scored significantly (p≤0.01) higher panel ratings. Microbiologically, sausages with different formulations were found to be within the acceptable limit up to the 15^th^ day of refrigerated storage.

**Conclusion:**

The study revealed that traditional hot smoking has slightly higher edges over the LS-treated sausages in terms of lipid oxidation, microbiological safety, and sensory panel ratings. However, if not superior, the same was found to be well within the acceptable limit in case of LS-treated sausages proving the potentiality of the use of LS as a suitable replacement for the traditional hazardous hot smoking process.

## Introduction

In the diverse range of value-added meat products, smoked meat products have always occupied a very important place due to their unique flavor and taste. Most of the peoples of North Eastern Region (NER) of India belong to various tribes/ethnic communities, and there are numbers of smoked meat recipes prepared and relished by these tribes. Smoking may be defined as the process of penetration of meat products by volatiles resulting from the thermal combustion of wood [1]. Smoking gives a drying effect to the meat, imparts desirable taste, brings out the color of the meat, and also retard the development of oxidative rancidity and the spoilage due to microbial invasion [2]. All these beneficial effects of smoking on meat may be attributed to the combined effects of antimicrobial and antioxidative activities of formaldehyde, carboxylic acids, and phenols [3]. Development of desired sensory properties (such as color, flavor, and appearance) together with safety of the product is main concern in the traditional method of smoking [4].

Although smoking renders the meat products shelf stable for a sufficient length of time and increases its palatability, from scientific studies, it has been concluded that frequent consumption of smoked meat products may also lead to the development of carcinoma of gastrointestinal tract due to the presence of carcinogenic polycyclic aromatic hydrocarbons (PAHs) called benzo(a)pyrene [5]. About 660 different compounds belonging to the PAH group have been identified so far [6] and as reported, these are the largest class of chemical compounds known to be cancer-causing agents [7]. As conventional smoking (CS) leads to the formation of carcinogenic PAHs, the application of liquid smoke (LS) has been found to be a very promising alternative in preserving and flavoring different meat products without any health hazard [5]. Moreover, there are various advantages of the application of LS over the traditional smoking process such as controlled and even distribution of the flavor, environment-friendly, and wider options of application such as dipping, spraying, and integrated mixing [8].

Keeping in view the importance of smoked meat products in the culinary practices of consumers of NER, it was decided to study the effect of conventional hot smoking as well as the application of LS at different levels on the nutritional, physicochemical, microbiological, and sensory qualities of pork sausages.

## Materials and Methods

### Ethical approval

Ethical approval was obtained from the Institutional Animal Ethics Committee, Faculty of Veterinary Science, AAU, Khanapara, Guwahati - 781 022.

### Materials

Fresh pork procured from the local market was carried hygienically to the laboratory and washed thoroughly. After washing, the meat was deboned manually, and all the separable fats were removed from the lean portion along with visible fascia and cartilages. The lean pork and the fat were cut into small chunks, vacuum packed in high-density polyethylene bags and stored in a deep freezer maintained at −18°C till used. Best quality spices available in the local market were purchased, washed, sun dried, and ground were necessary for using the same in sausage formulations. The condiment paste comprising ginger, garlic, and onion was prepared afresh for each batch of sausages.

Cellulose casing (21 mm diameter) available in the department was utilized for preparation of the sausages, while liquid smoke utilized in the present study was procured from M/s. Red Arrow International, Manitowoc, USA. The concentrated LS was diluted (V/V) at 0.5% with sterile distilled water to prepare the stock solution. On the basis of a pilot scale study, three different concentrations of the stock solution, i.e., 3%, 5%, and 7% were selected for the final study.

### Methods

#### Preparation of sausages

The pork stored at −18°C was first thawed overnight in a refrigerator maintained at 6±1°C and then minced twice in a mechanical meat mincer having a sieve diameter of 4 mm. The minced pork was then cured for 24 h at refrigeration temperature 6±1°C. Next day, the sausage emulsion was prepared by mixing the required amount of fat, non-meat ingredients, spices, and condiments as per a basic recipe in a bowl chopper. The emulsion thus prepared was divided into five parts. The control emulsion (C) and the emulsion for CS (T_1_) were stuffed directly into cellulose casings with the help of an electrically operated sausage stuffer (Make: Sirman, Model: IS V25 IDRAVERT). However, the other three parts of emulsions (T_2A_, T_2B_, and T_2C_) were thoroughly mixed with 3%, 5%, and 7% diluted LS, respectively, before stuffing into the cellulose casings. Thereafter, the green sausages from each of the formulations were cooked in a cooking vat (Make: Talsabell) maintained at 75°C for 45 min. Subsequently, the cooked control (C) and the LS-treated sausages (T_2A_, T_2B_, and T_2C_) were allowed to cool down to room temperature, and vacuum packed. However, the cooked sausages intended for hot smoking (T_1_ formulation) process were introduced to the smoke chamber maintained at 65°C for 45 h with 80-90% relative humidity [9] and vacuum packed soon after cooling to room temperature. Thereafter, the sausages with different treatments were analyzed for nutritional, physicochemical, microbiological, and sensory attributes.

#### Proximate composition

Proximate composition of the sausages in terms of percent moisture, crude protein (CP), ether extract (EE), and total ash was determined on the day of production [10] (Association Official Analytical Chemists, 1995).

#### Physicochemical properties

Emulsion stability (ES)

ES of the sausage emulsion was determined by the method as described by Mongale *et al*. [11] before stuffing into casings.

% Cooking loss (CL)

The percentage of CL was calculated on the day of production as per the method given by El-Nashi *et al*. [12].

pH

The pH of the sausages was determined by following the method as described by Pippen *et al*. [13] using a digital pH meter (Make: Metrohm, Model: 801 Stirrer) on the day of production.

Water activity (a_w_)

The water activities of the ready-to-eat sausages were determined on the 1^st^ day of the experiment with the help of a_w_ meter (Make: AQUA LAB, Model: 4TE).

Thiobarbituric acid reactive substance (TBARS) value

TBARS values of the sausages were determined at different periods of storage as per the standard method given by Witte *et al*. [14].

#### Objective sensory qualities

Shear force analysis

The shear force values of the sausages soon after preparation were determined using the Food Texture Analyzer (Make: Stable Micro Systems, Model: TA-HD plus) with the help of Warner-Bratzler blade set. The sausages samples were allowed to cut into three different places, and the force (in kg) required to cut through was recorded. The average of all the three readings was recorded as the shear force in kg.

Texture profile analysis (TPA)

The texture profile of the sausages in terms of hardness, fracturability, springiness, cohesiveness, chewiness, and resilience was evaluated using the food texture analyzer (Make: Stable Micro Systems, Model: TA-HD plus) using the aluminum cylindrical probe (SMSP/36R). Well-tempered sausage samples of uniform size were placed on the heavy duty platform and allowed for 50% compression. The samples were compressed twice during the test with the pre-test speed of 1 mm/s, test speed of 2.0 mm/s, and the post-test speed of 10.0 mm/s.

#### Organoleptic qualities

Ready-to-eat sausages treated with different methods of smoking were subjected to evaluation for organoleptic qualities by serving them to a semi-trained panel of nine members. All the samples were evaluated for appearance, color, flavor, texture, juiciness, and overall acceptability using a 7-point hedonic scale as described by Bratzler [15].

#### Microbiological quality

Enumeration of the total viable plate count (TVPC) and the total psychrophilic count (TPC) (log colony-forming unit [CFU]/g) of the sausage samples was done at 5 days interval up to 15 days in standard plate count agar medium as described by Harrigan and McCancy [16] and incubated at 37°C and 6±1°C for up to 24 h, respectively. However, the yeasts and molds (Y and M) count of the sausage samples was made on Rose Bengal Chloramphenicol Agar at similar time intervals (as in case of TVPC and TPC) and incubated at 37°C up to 4 days.

### Statistical analysis

The data obtained on studying different parameters were analyzed statistically by employing the SAS, Version 2 software. A total of five batches of sausages with different formulations were prepared for the present study.

## Results and Discussion

### Proximate composition

In terms of proximate composition (Table-1) of the ready-to-eat pork sausages, the highest percentage moisture (p≤0.01) was recorded by sausages of T_2C_ formulation, and it may be due to the addition of the highest amount of LS (7%) in the emulsion. Yusnaini *et al*. [17] also reported increase in the percentage moisture of LS immersed beef with increase in levels of LS. There was a significant reduction (p≤0.01) in the moisture content of sausages of T_1_ formulation which may be attributed to the drying effect of CS on the sausages [2]. Likewise, Swanepoel *et al*. [18] also reported that hot smoked pork products had lower moisture content than that of non-smoked products.

**Table-1:** Effect of CS and LS on proximate composition (%) of pork sausages (mean±SE).

Treatment	Moisture (%)	CP (%)	EE (%)	TA (%)
C	61.27±0.50^A^	18.51±0.47	18.03±0.79^A^	1.29±0.12
*T_1_	55.31±0.28^B^	19.12±1.28	21.66±0.98^B^	1.29±0.06
T_2A_	62.17±0.36^A^	19.57±0.54	16.16±0.30^AC^	1.23±0.09
T_2B_	62.38±0.32^A^	18.66±0.23	16.91±0.51^AC^	1.13±0.06
T_2C_	64.16±0.31^C^	18.39±0.34	15.02±0.38^C^	1.20±0.09

n=5, p≤0.01, CS=Conventional smoking, LS=Liquid smoke. Means having no superscript column wise (capital letter) do not differ significantly. C=Control,

* T_1_=Conventionally smoked, (T_2A_, T_2B_, and T_2C_)=Liquid smoke treated. CP=Crude protein, SE=Standard error, EE=Ether extract, TA=Total ash

The percentage EE content of the T_1_ formulation of sausages recorded significantly higher (p≤0.01) values than the rest of the formulations which may be due to highest CL and also due to loss of moisture content in the smoking process [19]. Choi *et al*. [20] reported that loss of moisture by a smoking process resulting in higher fat content of the restructured sausages. However, there was a gradual decrease (p≥0.05) in the percentage EE content of the LS-treated sausages which may be due to a gradual increase in their corresponding moisture contents. Similar results regarding the effect of LS on fat content of the beef sample were obtained by Yusnaini *et al*. [17]. They reported fat content of LS immersed beef tend to decrease on every level of dilution. During the study, no difference (p≥0.05) was noted in respect of percentage CP and percentage TA content of the sausages, indicating that smoking process has very minor effect on the protein and mineral content of sausages since the core formulation for the recipe was the same for each treatment groups.

### Physicochemical qualities

ES (ml of oil/100 g emulsion) of the control (Table-2) formulation was recorded to be superior in comparison to other formulations. ES revealed a significantly increasing trend with the application of increased amount of LS in the T_2A_, T_2B_, and T_2C_ formulations. LS may be oil based or water based [21], and as the LS used in the present study was oil based, this may be reason behind the release of more amount of oil in LS-treated formulations, leading to poor ES.

**Table-2:** Effect of CS and LS on ES, % CL, pH, a_w_, and shear force values (mean±SE) of pork sausages.

Parameters	Control	T_1_	T_2A_	T_2B_	T_2C_
ES (ml of oil/100 g)	1.88±0.12^A^	1.88±0.12^A^	2.20±0.12^A^	2.64±0.22^B^	3.20±0.10^C^
%CL	5.58±0.46^A^	16.42±0.52^B^	5.79±0.37^CA^	7.03±0.20^C^	6.46±0.44^CA^
pH	5.88±0.08^A^	5.45±0.09B	5.91±0.14^A^	5.97±0.07^A^	5.77±0.17^AB^
a_w_	0.986±0.004	0.979±0.002	0.983±0.003	0.986±0.005	0.988±0.004
Shear force (Kg F)	0.608±0.088^A^	1.023±1.75^B^	0.671±0.093^A^	0.555±0.065^A^	0.566±0.072^A^

n=5, p≤0.01, CS=Conventional smoking, LS=Liquid smoke. Means having no superscript row wise (capital letter) do not differ significantly. C=Control, *T_1_=Conventionally smoked, (T_2A_, T_2B_, and T_2C_)=Liquid smoke treated. ES=Emulsion stability, CL: Cooking loss, a_w_=Water activity, SE=Standard error

In terms of percentage CL (Table-2), the sausages of the T_1_ formulation recorded the highest (p≤0.01) where both cooking and CS process was applied. This may be due to high moisture loss as a result of application of both cooking and smoking. Higher temperature may cause protein denaturation and a considerable lowering in water holding capacity [22]. The results of the present study corroborate well with the findings of Kim *et al*. [3] who reported that percentage CL of smoked pork sausage was higher due to extensive thermal processing and the resultant loss of moisture from the meat. Similar findings were also reported by Dharmaveer *et al*. [19] who evaluated the effect of CS on chevon sausages.

The pH (Table-2) of the conventionally smoked sausages (T1) recorded soon after production exhibited significantly (p≤0.01) lower values than rest of the formulations which could be due to the production of various organic acids during the process of smoking [23]. Deuri *et al*. [24] and Choi *et al*. [20] also reported that smoking could effectively decrease the pH values of smoked pork products and restructured pork sausages, respectively. The sausages with different treatments did not reveal (p≥0.05) any difference in terms of mean a_w_ values as recorded on the day of production. However, the T_1_ sausages recorded the lowest a_w_ (Table-2) among the finished sausages. This might be due to loss of water from the T_1_ sausages which were first cooked and then smoked [22].

The results obtained in the study in respect of TBARS values (mg malanoldehyde [MDA]/Kg) revealed a progressive increase (p≤0.01) in all the treatment groups (Table-3) throughout the study period of 15 days ranging from 0.11 to 0.33 mg MDA/Kg. However, significantly lower (p≤0.01) levels of thiobarbituric acid reactive substance (TBARS) values were recorded in the treated sausages which might be due to the antioxidative effect of various phenolic compounds present in conventional and LS [2,3] and also due to vacuum packaging of sausage samples [25]. At the end of the storage periods, the TBARS values of all the sausage samples were recorded to be well within the permissible limits of 1-2 mg MDA/Kg [26]. The results of the present study corroborate well with Schwert *et al*. [27] who studied the liquid and traditional smoke on oxidative stability, color, and sensory properties of Brazilian Calabrese sausage.

**Table-3:** Effect of CS and LS on TBARS values (mg MDA/kg) of pork sausages at different storage periods (mean±SE).

Treatment	TBARS			

	1^st^ day	5^th^ day	10^th^ day	15^th^ day
C	0.12±0.002_a_	0.21±0.004_b_^A^	0.28±0.002_c_^A^	0.33±0.01_d_^A^
*T_1_	0.11±0.003_a_	0.19±0.001_b_^B^	0.24±0.002_c_^B^	0.25±0.002_d_^B^
T_2A_	0.10±0.02_a_	0.19±0.002_b_^B^	0.21±0.001_c_^C^	0.24±0.001_d_^B^
T_2B_	0.11±0.004_a_	0.18±0.001_b_^CB^	0.22±0.001_c_^D^	0.26±0.007_d_^CB^
T_2C_	0.10±0.003_a_	0.19±0.002_b_^B^	0.22±0.002_c_^D^	0.24±0.001_d_^B^

n=5, p≤0.01, CS=Conventional smoking, LS=Liquid smoke. Means having no superscript column wise (capital letter) and subscript row wise (small letter) do not differ significantly. C=Control,

* T_1_=Conventionally smoked, (T_2A_, T_2B_, and T_2C_)=Liquid smoke treated, SE=Standard error, MDA=malanoldehyde

### Shear force and TPA

The highest (p≤0.01) shear force values (Table-2) among all the sausage formulations were recorded by the hot smoked (T_1_ formulation) sausages. The significantly higher (p≤0.01) shear force values recorded in T_1_ formulation might be due to loss of moisture from the sausage during cooking and subsequent smoking. The findings of the present study corroborate well with Dharmaveer *et al*. [19] who reported increased hardness of chevon sausages due to a reduction in the moisture content by smoking. Similar findings were also reported by Kim *et al*. [3] and Awonorin [28].

In this study, there was no significant difference (p≥0.05) recorded in terms of TPA parameters (Table-4) of sausages with different treatments. However, the highest scores in terms of hardness, chewiness, cohesiveness, and resilience were recorded by the hot smoked sausages (T_1_ formulation). Similar reports were also made by Martinez *et al*. [29] who studied the effects of two commercial LS flavorings on the texture of salted pork loin and salted bacon and reported lowest values for hardness, fracturability, cohesiveness, gumminess, and chewiness for bacon treated with LS.

**Table-4:** Effect of CS and LS on texture profile of pork sausages (mean±SE).

Treatment	Hardness (N/cm^2^)	Fracturability (N)	Chewiness (N/cm)	Cohesiveness (ratio)	Springiness (cm)	Resilience (cm)
C	2.761±0.686	8.511±2.712	0.808±0.214	0.455±0.012	0.575±0.027	0.210±0.008
*T_1_	4.458±0.416	7.520±1.676	0.996±0.117	0.490±0.030	0.489±0.023	0.243±0.035
T_2A_	3.319±0.670	7.131±0.989	0.816±0.205	0.452±0.015	0.508±0.030	0.218±0.013
T_2B_	3.285±0.614	7.591±1.002	0.907±0.244	0.437±0.024	0.593±0.044	0.210±0.022
T_2C_	2.812±0.578	8.051±1.499	0.657±0.220	0.432±0.036	0.517±0.037	0.203±0.027

n=5, p≥0.05, CS=Conventional smoking, LS=Liquid smoke. Means having no superscript column wise (capital letter) do not differ significantly. C=Control,

* T_1_=Conventionally smoked, (T_2A_, T_2B_, and T_2C_)=Liquid smoke treated, SE=Standard error

### Microbiological quality

In the present study, significant differences (p≤0.01) in terms of TVPC (log CFU/g) among the control and treated sausages (Table-5) on various days of preservation were observed. TVPC counts were below the countable range at the beginning of the study which might be due to the hygienic processing of the sausages and low initial contamination of the product [23]. From the 5^th^ day onward, all the formulations depicted a gradual increase in numbers of viable organisms; however, the treated sausages recorded significantly lower (p≤0.01) counts in comparison to control sausages. This might be due to the antibacterial effects of hot smoking process and LS [24,30,31]. Moreover, the vacuum packaging employed in the present study might influence lower counts for viable organisms in all the formulations.

**Table-5:** Effect of CS and LS on TVPC (log CFU/g) of pork sausage at different storage periods (mean±SE).

Treatment	1^st^ day	5^th^ day	10^th^ day	15^th^ day
C	<25	3.23±0.01^A^	4.83±0.05^A^	5.43±0.06^A^
*T_1_	<25	<25	3.33±0.01^B^	4.17±0.02^B^
T_2A_	<25	3.21±0.01^A^	4.35±0.03^C^	5.24±0.04^C^
T_2B_	<25	3.13±0.01^B^	4.39±0.02^C^	5.31±0.01^C^
T_2C_	<25	3.07±0.01^C^	4.29±0.02^C^	5.23±0.01^C^

n=5, p≤0.01, CS=Conventional smoking, LS=Liquid smoke. Means having no superscript column wise (capital letter) do not differ significantly. C=Control,

* T_1_=Conventionally smoked, (T_2A_, T_2B_, and T_2C_)=Liquid smoke treated. TVPC=Total viable plate count, CFU=Colony-forming unit, SE=Standard error

In respect of mean TPC counts (log CFU/g) of the sausage samples (Table-6), hot smoked sausages recorded a growth below the countable range in all days of observation. This might be due to the superior antimicrobial properties of CS process on the pork sausages [2]. Although there was a gradual increase in the mean TPC count in the control and LS-treated sausage samples throughout the storage period, the counts in respect of LS-treated sausages were significantly lower (p≤0.01) than the control sausages. This may be due to the effect of LS which contains different antimicrobial substances [32].

**Table-6:** Effect of CS and LS on TPC (log CFU/g) of pork sausage at different storage periods (mean±SE).

Treatment	0 day	5^th^ day	10^th^ day	15^th^ day
C	<25	<25	2.40±0.03^A^	3.34±0.02^A^
*T_1_	<25	<25	<25	<25
T_2A_	<25	<25	2.44±0.02^A^	3.31±0.01^A^
T_2B_	<25	<25	2.31±0.01^B^	3.31±0.02^A^
T_2C_	<25	<25	2.27±0.01^B^	3.21±0.01^B^

n=5, p≤0.01, CS=Conventional smoking, LS=Liquid smoke. Means having no superscript column wise (capital letter) do not differ significantly. C=Control,

* T_1_=Conventionally smoked, (T_2A_, T_2B_, and T_2C_)=Liquid smoke treated. TPC=total psychrophilic count, CFU=Colony-forming unit, SE=Standard error

The hot smoked sausages did not reveal any growth of Y and M colonies for the entire periods of study. This phenomenon may again be attributable to the superior antimicrobial effect of hot smoking process on the target food product [2,33,34]. Comparable findings were also reported by Özer *et al*. [35], who could not detect any Y and M colonies in the vacuum packed thornback ray sausages stored at chilled conditions treated with hot smoking process. There was a gradual but non-significant (p≥0.05) increase in the Y and M counts (Table-7) in the control and LS-treated sausage samples on the 10^th^ and 15^th^ days of refrigerated storage. However, apparently lower counts of Y and M as recorded in LS-treated samples may be attributed to the inhibitory effect of LS on Y and M counts of sausages. Akin findings were also made by Morey *et al*. [36] who studied the effect of LS on the quality attributes of frankfurters and reported that the Y and M counts were <1 log CFU/g or below throughout the study.

**Table-7:** Effect of CS and LS on Y and M count (log CFU/g) of pork sausage at different storage periods (mean±SE).

Treatment	0 day	5^th^ day	10^th^ day	15^th^ day
C	ND	ND	0.99±0.60	1.37±0.84
*T_1_	ND	ND	ND	ND
T_2A_	ND	ND	0.91±0.56	1.35±0.82
T_2B_	ND	ND	1.14±0.70	1.33±0.816
T_2C_	ND	ND	1.08±0.66	1.25±0.76

n=5, p≥0.05, CS=Conventional smoking, LS=Liquid smoke. Means having no superscript column wise (capital letter) do not differ significantly. C=Control,

* T_1_=Conventionally smoked, (T_2A_, T_2B_, and T_2C_)=Liquid smoke treated, Y&M=Yeast and Mold, CFU=Colony-forming unit, SE=Standard error

### Sensory evaluation

**Figure-1:**
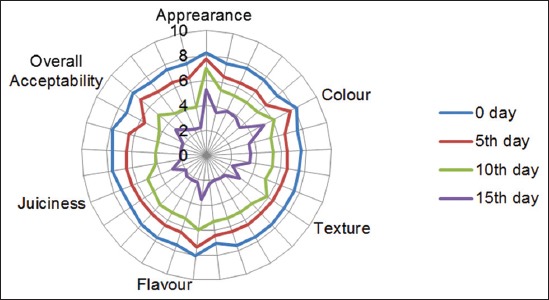
Effect of CS and LS on sensory qualities of pork sausage at different storage periods CS=Conventional smoking, LS=Liquid smoke.

In the present study, irrespective of formulations, all the sausage samples exhibited a gradual declining of sensory scores throughout the study period. However, the mean sensory scores in respect of all the eating quality attributes were found to be significantly higher (p≤0.01) for hot smoked sausages (Figure-1) in comparison to other treatments including control. Formation of typical smoke color on the surface of the sausages may be the reason behind higher (p≤0.01) panel rating in respect of appearance and color of the hot smoked sausages. Similar findings were also reported by Deuri *et al*. [24] who studied the effect of curing ingredients and vacuum packaging on the physicochemical and storage quality of ready-to-eat smoked pork product (*Vawksa rep*). Flavor of meat product is generally influenced by the lipid oxidation, and in the present study, the flavor intensity of the sausages treated with different methods of smoking revealed a gradual decrease in the mean flavor scores with the exception in case of hot smoked sausages, which recorded significantly higher (p≤0.01) mean flavor scores in all the days of storage study. This may be attributed to corresponding low TBARS values of the T_1_ sausages during the entire storage period [2]. Fat contributes to juiciness by sustained stimulation of salivary glands and release of saliva while chewing [37], and in the present study, the higher (p≤0.01) juiciness in case of hot smoked sausages may be due to its higher percentage EE content. It was observed that the irrespective of treatments applied, all the sausages recorded gradual loss of texture scores during the period of storage and this may be due to consequent loss of moisture from the sausage samples during storage. However, the hot smoked sausages enjoyed a higher texture (p≤0.01) score throughout the study which may be due to higher mean fat content which has a major effect on texture, juiciness, mouth feeling, and flavor of the meat products [38]. In terms of mean overall acceptability scores, the hot smoked sausages recorded the highest scores throughout the study period while the control samples scored lowest panel ratings on the 15^th^ day of evaluation. The higher acceptability in case of hot smoked sausages may be due to corresponding higher scores in all the sensory traits by this group of sausages. Similar reports in terms of overall acceptability were also reported by Choi *et al*. [39] in smoked meat products.

## Conclusion

Based on the results of various parameters studied in the present investigation, it may be concluded that though hot smoked sausages (T1 formulation) were found to be superior in terms of the prevention of lipid oxidation, microbiological, and sensory indices, LS-treated sausages (T_2A_, T_2B_, and T_2C_) could maintain the same to a desirable level up to the 15^th^ day of refrigerated storage. Therefore, keeping in view the association of hazardous PAHs compounds with hot smoking of meat and meat products, application of LS in preparation seems to be very promising and needs to be exploited by the entrepreneurs for marketing healthier meat products.

## Authors’ Contributions

DB carried out the product preparation part, AD prepared the background of the research and prepared the manuscript, SKL and DPB performed laboratory evaluation of samples, ST performed the collection and analysis of data, and MH revised the manuscript. All the authors have read and approved the final manuscript.
